# In Vitro Study of Nitric Oxide Metabolites Effects on Human Hydatid of *Echinococcus granulosus*

**DOI:** 10.1155/2009/624919

**Published:** 2009-12-13

**Authors:** Razika Zeghir-Bouteldja, Manel Amri, Saliha Aitaissa, Samia Bouaziz, Dalila Mezioug, Chafia Touil-Boukoffa

**Affiliations:** Laboratoire de Biologie Cellulaire et Moléculaire, FSB-USTHB, Université Bab-Ezzouar, Bp 32, 16111 Algiers, Algeria

## Abstract

Hydatidosis is characterized by the long-term coexistence of larva *Echinococcus granulosus* and its host without effective rejection. Previous studies demonstrated nitric oxide (NO) production (in vivo and in vitro) during hydatidosis. In this study, we investigated the direct in vitro effects of NO species: nitrite (NO_2_^−^), nitrate (NO_3_^−^) and peroxynitrite (ONOO^−^) on protoscolices (PSCs) viability and hydatid cyst layers integrity for 24 hours and 48 hours. Our results showed protoscolicidal activity of NO_2_^−^ and ONOO^−^ 24 hours and 3 hours after treatment with 320 *μ*M and 80 *μ*M respectively. Degenerative effects were observed on germinal and laminated layers. The comparison of the in vitro effects of NO species on the PSCs viability indicated that ONOO^−^ is more cytotoxic than NO_2_^−^. In contrast, NO_3_^−^ has no effect. These results suggest possible involvement of NO_2_^−^ and ONOO^−^ in antihydatic action and point the efficacy of these metabolites as scolicidal agents.

## 1. Introduction

Hydatidosis is a chronic infection of medical and veterinary importance caused by the larval stage of cosmopolitan parasitic platyhelminth *Echinococcus granulosus. *This disease is considered as re-emerging zoonosis in several countries [1] and endemic in Algeria; it is considered as disease for obligatory declaration by the National Institute of Public Health (INSP). Although surgery is the main therapeutic approach, medical and percutaneous treatments have been used in recent years [2]. Currently, benzimidazole compounds (albendazole and mebendazole) are used in medical treatment [3]. Percutaneous treatment and injection with scolicidal compounds has become an alternative to the surgery because of successful results obtained in recent studies [4, 5]. 

Larval forms develop into large fluid-filled cysts in intermediate hosts. The hydatid cyst consists of two layers: an inner germinal layer and an outer carbohydrate-rich laminated acellular layer which protects the parasite from host immune cells [6, 7]. The germinal layer (GL) is cellular. It gives through budding and differentiating the infective protoscolices (PSCs). Cysts containing these larval forms are considered to be fertile. Hydatid cyst walls are important in the establishment and persistence of infection [8].

Nitric oxide (NO) has been postulated to play a role in the host defence mechanism in hydatidosis [9, 10]. We have previously shown the production of NO_2_^−^ in sera and cystic fluids obtained from patients carrying different cystic localization. Furthermore, Human inducible NO synthase (NOS2) expression during hydatidosis has been detected in liver biopsies from patients by immunochemical method. NOS2 expression was observed in hepatocytes and Kuppfür cells from hydatid patients [9, 11]. 

NO is an important regulator and mediator in many physiological and pathophysiological events. It is synthesized by a family of NOS isoforms using L-arginine as the substrate. It has been implicated in neurotransmission, vasodilatation, and immune regulation [12]. In response to stimuli, activations of NADPH oxidase and NOS2 contribute to macrophage-mediated pathogen killing [13]. Consequently, activated macrophages express NOS2 to produce copious amounts of NO. This high NO production has been implicated in several cytostatic and cytotoxic actions against a number of pathogens, owing to the radical nature of NO. NO is very reactive toward oxygen; it yields nitrite (NO_2_^−^) and nitrate (NO_3_^−^) as end products. The simultaneous production of superoxide anion (O_2_^−^) and NO combines at diffusion controlled rates to produce peroxynitrite (ONOO^−^) [14]. It is more potent oxidant and cytotoxic mediator than NO or superoxide alone [15]. Peroxynitrite oxidizes and nitrates a variety of targets; it was received much attention as the potential mediator of NO cytotoxic effects. Contribution of ONOO^−^ to microorganisms killing has been also proposed. Antiparasitic effect of NO has been reported on protozoan parasites (*Leishmania, Trypanosoma, Entamoeb*a)* and* metazoa including *Schistosoma, Fasciola, and Echinococcus.*

Despite the production of NO in the host response to *E. granulosus* infection, direct effects of NO metabolites (NO_3_^−^, NO_2_^−^, and ONOO^−^) on parasite survival are not investigated. The present study is designed to test the efficacy of these metabolites on the viability of PSCs and cystic wall of human hydatid cysts in vitro. Using culture system of PSCs and pieces of cystic walls, we investigated the effects of (NO_3_^−^, NO_2_^−^, ONOO^−^) on PSCs tegument and cystic walls integrity. The morphological changes are important in indicating the efficacy of metabolites because the hydatid cyst walls constitute the interface between the macroparasite and hosts. The time of incubation is an important factor in defining the susceptibility of the parasites to the action of metabolites in vitro and might be a promising protoscolicidal agent in hydatid surgery.

## 2. Materials and Methods

### 2.1. Parasite Materials

*Twelve E. granulosus *hydatid cysts were obtained from hydatid patients who carried hepatic and pulmonary cysts after surgical extirpation. All patients were admitted to the Mustapha Bacha hospital (Department of surgery, Algiers, Algeria). The mean age of the patients was 33 ± 2.5 years. They did not present other parasitic or bacterial infections. None of the patients had received pharmacological treatment. All subjects are informed consent for the present study, which was carried out according to the guidelines of the local Ethics Working Group. Cyst fertility was determined by the presence of free protoscolices (PSCs) in cystic fluid. Other parameters of cyst fertility are considered such as a whitish color of laminated layer and limpidity of cystic fluid.

### 2.2. Peroxynitrite Synthesis

Peroxynitrite was synthesized from sodium nitrite and hydrogen peroxide using a quenched flow method and assayed spectrophotometrically at 302 nm (Extinction coefficient = 1670 M^−1^cm^−1^). Solution of peroxynitrite was stored at −80°C [14, 16].

### 2.3. Protoscolices Preparation

After aseptic dissection of the intact hydatic cysts, the fluid was removed and centrifuged at 3000 rpm for 30 minutes at 4°C. The pellet containing PSCs was washed resuspended in PBS (Phosphate Buffered Saline) pH 7.2 then adjusted and adjusted to 10^3^ PSCs/ml in RPMI1640 medium supplemented with 15 mM Tris HCl pH 7.5, 2 mM glutamine, and 10% fetal calf serum. The viability of PSCs was confirmed prior to the experiments. It was determined by body movement observed under inversed microscopy and vital staining with 0.1% methylene blue. All samples had a viability >98% at the time of culture.

### 2.4. Cyst Layers Preparation

Germinal layer (GL) joined to laminated layer (LL) was dissected from open intact cysts. Portions of these membranes were cut and washed three times in PBS pH 7.2. The germinal layer was carefully scraped from the laminated layer.

### 2.5. Protoscolices and Pieces of Cystic Walls Cultures

Protoscolices and pieces of germinal and laminated layers were cultured in RPMI1640 medium supplemented with 10% fetal calf serum (FCS). They were cultured in the absence and presence of increasing concentration of sodium nitrite (NaNO_2_), sodium nitrate (NaNO_3_), and Sodium peroxynitrite (NOONa) (20, 40, 80,160 and 320 *μ*M) incubated at 37°C in humid atmosphere of 5% CO_2_ for 24 hours and 48 hours. The morphological changes were observed under microscope with inversed phase.

### 2.6. Statistical Analysis

PSCs cultures were performed in triplicate for each incubation conditions. All values are expressed as means ± standard deviation (SD). Data analysis was performed using the Origin Pro.Lab. Differences between means were analysed by ANOVA (analysis of variance). Values for *P* < .05 were considered statistically significant.

## 3. Results

### 3.1. Effects of Nitric Oxide Metabolites on Protoscolices (PSCs) Viability

**Figure 1:**
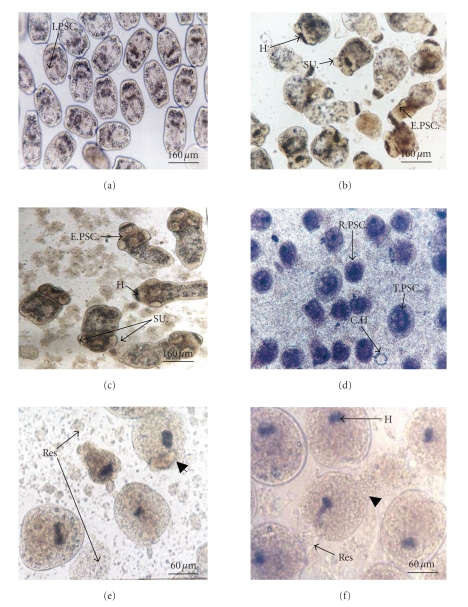
Morphological aspects of cultured PSCs in presence of exogenous nitric oxide metabolites. (a) Cultured invaginated PSCs at *t* = 0 (initial PSCs viability = 98%); (b) cultured PSCs after 48 hours of incubation without any treatment, (c) after 48 hours of incubation with 320 *μ*M of NO_3_^−^, (d) after 24 hours of incubation with 320 *μ*M of NO_2_^−^, (e) after 3 hours of incubation with 80 *μ*M of ONOO^−^, and (f) 1 hour of incubation with 160 *μ*M of ONOO^−^. (H): Hooks; (CH): Crown of hooks; (SU): Suckers; (I.PSC): invaginated protoscolices; (E.PSC): evaginated protoscolices; (T. PSC): Turgescent protoscolices; (R.PSC): retracted protoscolices; (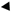): Alteration of tegument; (Res): Residues of dead protoscolices.

Nitrate was not effective on PSCs viability. This observation was confirmed by statistical study (ANOVA) for 24 hours and 48 hours compared with the control culture. It has no significant differences between the mortality of cultured PSCs in presence of increasing concentrations of nitrate for 24 hours (*P* = .99) and 48 hours (*P* = .993) compared to control culture (Table 1). The morphology of PSCs after 24 hours of treatment remained largely undisrupted. Microscopic examination showed that majority of PSCs were still viable; they exhibited distinct movements and conserved with conserv the membrane integrity, order of hooks, and calcareous corpuscles. Of those, some were invaginated, others were evaginated, and suckers were clearly visible (Figure 1(c)).These observations indicate the morphological evolution of PSCs in culture medium in spite of treatment with nitrate.

Loss of PSCs viability in nitrite-treated cultures became after 24 hours with 71.14 ± 2.39% of mortality in presence of 320 *μ*M of NO_2_^−^ (Table 1). After 48 hours, we observed that scolicidal activity was affected by nitrite in concentration dependent manner. 

Microscopic examination of treated parasites showed distortion of the PSCs morphology and degenerative effect. This effect is characterized by loss of motility compared to the untreated PSCs. We observe with interest turgescence of some PSCs and retractability of others. This tegumental disruption concerned the both forms of invaginated and evaginated PSCs (Figure 1(d)). Alteration of PSCs structure showed disruption of external plasma membrane of soma (caudal region) with liberating vesicles and calcareous corpuscles in culture medium. The presence of free hooks in culture medium indicated their detachment and destruction of the tegument (Figure 1(d)).

A significant reduction in motility was observed after few minutes in cultures treated with 80 *μ*M of peroxynitrite .The PSCs viability is dramatically affected after 20 minutes of incubation (100% of mortality) (Table 1). Loss of PSCs viability appeared after treatment with 40 *μ*M of peroxynitrite. The viability is reduced at 50% of viable parasite (Table 1).

The mortality of PSCs is significantly different between treated parasites with different concentrations of peroxynitrite after 24 hours of incubation at 37°C. Microscopic examination showed no movement and we observed retraction of PSCs. The suckers are disappeared; they are not visible. The presence of residues of membranes (ghost membranes) indicates destruction of the tegument (Figures 1(e) and 1(f)).

### 3.2. Effects of Nitric Oxide Metabolites (Nitrite and Peroxynitrite) on Hydatid Membranes

**Figure 2:**
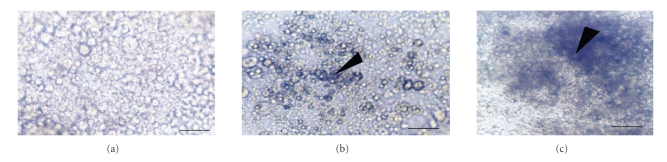
Morphological changes of germinal layer alone. (a) without any treatment, (b) with 320  *μ*M of NO_2_^−^ after 24 hours of incubation, and (c) with 80  *μ*M of ONOO^−^ after 20 minutes of incubation, (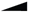): alteration of membrane. (bar = 30 *μ*m).

Incubation of isolated germinal layer (GL) from laminated layer (LL) with 80 *μ*M of peroxynitrite shows a high cytotoxic effect with alteration of the membrane integrity (Figure 2(c)) compared to the action of the nitrite (Figure 2(b)).

**Figure 3:**
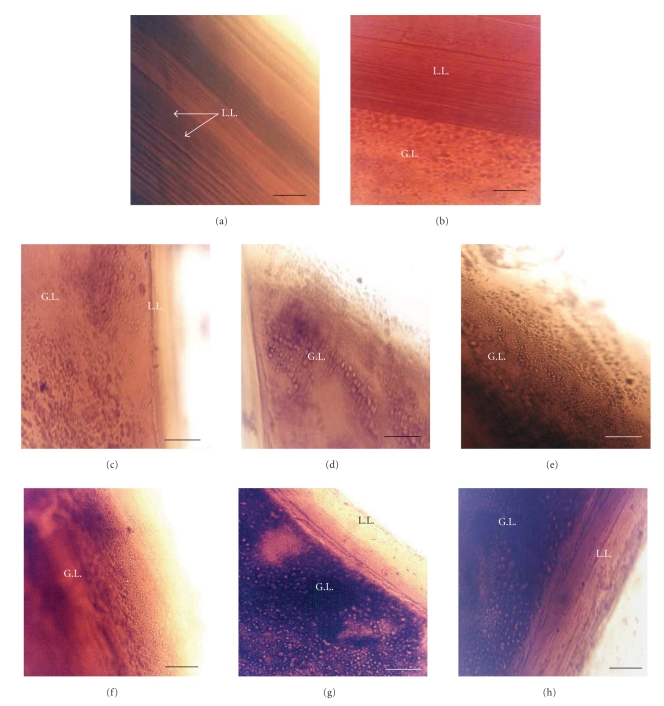
In vitro effects of exogenous nitric oxide metabolites on hydatid layers. (a) and (b) without any treatment; (c) treated with 160 *μ*M of NO_2_^−^ after 48 hours of incubation; (d) 320 *μ*M of NO_2_^−^ after 24 hours; (e) 320 of NO_2_^−^ *μ*M after 48 hours; (f) 80 *μ*M of ONOO^−^ after 15 minutes; (g) 80 *μ*M of ONOO^−^ after 15 minutes; (h) 160 *μ*M of ONOO^−^ after 20 minutes. (Bar = 50  *μ*m). (L.L): Laminated layer; (G.L): germinal layer.

Untreated laminated membrane (LL) is constituted of lamellar layer observed under microscope of inversed phase (see Figure 3(a)). The effects of nitrite are observed after 24 hours of incubation of layers with 320 *μ*M. These effects are characterized by brunishment of layers indicating degenerative effect of nitrite.

The first modifications related to the cytotoxic action of peroxynitrite appeared with time after 15 minutes of incubation in presence of 80 *μ*M of ONOO^−^. Loss of lamellar structure is also observed (Figures 3(g) and 3(h)). In vitro study of peroxynitrite toxicity on pieces of hydatid membranes has shown degenerative effects of peroxynitrite at 80 *μ*M. Intensity of cytotoxic action is characterized by darkening of germinal layer and pieces of joined layers. These modifications depend on the concentrations of the two metabolites and the time of exhibition (Figure 3).

## 4. Discussion

Cytotoxic effects of NO on hydatid cysts were previously investigated by determining the NO_2_^−^ levels in sera of hydatid patients and human cyst fluids (fertile and infertile) [11]. Furthermore, PBMCs from hydatid patients incubated with IFN-*γ* alone are effective in killing the PSCs in vitro [10, 17]. In the present work, the comparative study of the direct effects of NO metabolites suggests that reactive nitrogen intermediates (RNIs) play an important role in the antihydatic mechanism. Moreover, we have observed that the ONOO^−^ is a most effective and rapid scolicidal metabolite which may well be of clinical value than NO_2_^−^. Thus it may be possible to use this metabolite as the scolicidal agents in surgery to avoid the risk of dissemination of viable PSCs coming from fertile fluids and germinal layer that leading to secondary infection.

Based on these results, the cytotoxic effect of NO on PSCs viability and hydatid layers suggests the relevant role of NO in the host defense against hydatid infection. Our results are in agreement with most studies related to the antiparasitic role of NO. 

Endogenous and exogenous NO has been reported in inhibiting the development of intracellular parasites including *Trypanosoma, Leishmania, Plasmodium, Toxoplasma* and extracellular protozoa (*Entamoeba*) as well as *Schistosoma *[18]. It has been shown that apoptosis is induced in vitro by NO species in *Entamoeba histolytica* and renders them incapable of surviving in hamster's livers [19].

 Moreover, NO produced from activated peritoneal macrophages and S-nitroso-N-acetylpenicillamine has been reported in killing murine hydatid cysts of *Echinococcus granulosus *[20]. Moreover, NO produced from murine activated macrophages has been reported in killing PSCs of *Echinococcus multilocularis *in vitro [21]. The expression of the intracellular killing of *Leishmania major *amastigotes correlated with NO_2_^−^ production [22]. Interestingly, the therapeutic treatment of cutaneous leishmaniasis with NO-releasing drugs has been approached [23, 24].

In terms of the time course of NO_2_^−^ and ONOO^−^ action, our observation indicated that peroxynitrite is a rapid scolicidal metabolite but nitrite had a slow action. Both metabolites have a profound effect on the viability of PSCs. Disruption of the tegument and desorganization of the double crown of hooks are observed 24 hours after incubation of parasites with 320 *μ*M of NO_2_^−^; its cytotoxicity appeared to be a progressive process. Microscopic examination of NO_2_^−^-treated PSCs showed reduction in motility of PSCs. Furthermore, motility is an important characteristic of PSCs viability. Cytotoxic effect of NO has been detected by loss of body movement of parasite. On exposure to NO, *Brugia malayi* and *Onchocera linealis* showed reduction in motility within 5–30 minutes [25]. These results suggest a functional alteration of PSCs metabolism. 

Indeed, inactivation of parasite enzymes by NO appears to be relevant in inhibition of physiological functions that contribute to survival of parasite in the host and viability was affected. Inhibition has been suggested to explain the cytostatic effect of NO on *Trypanosoma brucei gambiense* and *T. b. brucei.* [26–28].

The present study is the first to demonstrate the in vitro cytotoxic effect of the ONOO^−^ on the hydatid viability (PSCs and cystic layers). This anion is probably implicated in the antihydatic mechanism in vivo. However, recent studies have demonstrated the ONOO^−^-mediated *Leishmania amazonensis* amastigote killing in vitro [29]. More ever, ONOO^−^ can decrease the life span of ovine liver flukes *Fasciola hepatica and Dicrocoelium dendriticum* in vitro [29]. ONOO^−^ is a potent oxidant implicated in a number of pathophysiological processes. Efficacy of ONOO^−^ is dictated to its reactivity and its diffusibility. In this study, the ONOO^−^ induces a dramatic effect on PSCs viability with loss of tegument integrity. Treated PSCs with 80 *μ*M of this anion showed rapid degenerative changes suggesting its usefulness in surgery as scolicidal agent (Figures 1(e) and 1(f)). ONOO^−^ induces cellular damage by triggering one of the basic cell death pathways, apoptosis or necrosis. ONOO^−^ initiates lipid peroxydation [30], causes DNA breakage [31], and reacts with thiols [30]. Peroxynitrite- induced protein modifications include protein oxidation and nitration. However, enzymes containing a redox active transition metal center are the prime targets of the oxidant [32]. Reactions of ONOO^−^ are affected by the local pH and the microenvironment with hydrophobic membrane compartments favoring nitration and aqueous environments favoring oxidation. Specifically, the reaction of carbon dioxide with peroxynitrite produces carbonate radical anion and nitrogen dioxide, whose concerted action leads to nitration of tyrosine residues forming nitrotyrosine, a marker of the toxic NO pathway. The detection of nitrotyrosine illustrates the site of peroxynitrite production and oxidative stress, providing evidence of the toxicity of NO [33].

In vivo studies of the leishmanicidal effect of peroxynitrite suggest that nitration/oxidation of parasite membrane proteins may be an important event. Functional alteration of membrane proteins may impair intracellular ionic composition and transport of essential metabolites, all of which are processes crucial to cell survival [29]. 

These data combined with our results suggest probable in vivo production of ONOO^−^ *during *hydatidosis and may be implicated in the host defense mechanism against *E. granulosus.*

This NO species is likely to be the main cytotoxic effector produced by the macrophages in vivo. For this, it is relevant to investigate cytotoxic effects of this anion in vivo during hydatidosis.

In addition to its efficacy against PSCs, ONOO^−^ also exhibits a considerable damaging effect on the hydatid membranes that characterized by darkening of the pieces of cystic layers. Germinal layer of* E. granulosus* had been shown also to be susceptible to the nitrite. In comparison of the cytotoxic effects of these two metabolites, we distinguish that the peroxynitrite is more cytotoxic than nitrite. After three days of incubation of pieces of cystic walls, we observe residues of germinal layer in culture medium isolated from laminated layer. 

The separation of the germinal layer (G.L.) from laminated layer (L.L.) deprives the first one to the source of nutriments essential in PSCs development, and this contributes to the nonviability of the hydatid, this may be a line to therapy approach. Prospectively, the introduction of NO donors could help patients suffering from cystic echinococcosis undergo improved chemotherapy by resorption of hydatid. 

In conclusion, with this comparison study, we provide here multiple lines of evidence for the role of NO and many other derived radicals in immune response against *E. granulosus*. The production of NO species by activated macrophages may be capable of a significant role in preventing the dissemination of *E. granulosus *infection. The results described here suggest the possible in vivo production of ONOO^−^ and its involvement in the antihydatid mechanism. In addition to the beneficial effect of NO, it can be detrimental to the host organs carrying the hydatid cysts. More human in vivo investigations are required to define the mechanisms by which peroxynitrite is cytotoxic.

## Figures and Tables

**Table 1:** Percentage of dead PSCs treated with increased concentrations of nitric oxide metabolites (NO_3_^−^, NO_2_^−^, ONOO^−^) after 24 hours and 48 hours of incubation (mean ± SD).

		Concentrations of nitric oxide metabolites (*μ*M)					

	Timeof incubation	Control 0	20	40	80	160	320

	24 hours	11,8 ± 40	11,3 ± 30	11,29 ± 5,41	11,45 ± 1,35	11,3 ± 2,00	11,65 ± 1,65
Nitrate	% of dead PSCs						
(NO_3_^−^)	48 hours	17,3 ± 0,7	17,65 ± 3,35	17,33 ± 2,67	17,41 ± 0,20	17,41 ± 2,15	16,40 ± 3,10
	% of dead PSCs						

	24 hours	3,85 ± 0,15	3,78 ± 0,21	3,92 ± 0,07	4,15 ± 0,15	4,3 ± 0,3	71,14 ± 2,29
Nitrite	% of dead PSCs						
(NO_2_^−^)	48 hours	5,25 ± 0,55	12,94 ± 0,94	23,07 ± 0,07	29,20 ± 0,20	64,98 ± 3,44	100
	% of dead PSCs						

Peroxynitrite	24 hours	6,01 ± 3,61	12,03 ± 7,23	50	100	100	100
(ONOO^−^)	% of dead PSCs						

## Abbreviations

NO: nitric oxide
IFN-*γ*: interferon-gamma
PBMCs: peripheral blood mononuclear cells
PSCs: Protoscolices
NO_3_^−^: nitrate
NO_2_^−^: nitrite
ONOO^−^: peroxynitrite
NOS2: nitric oxide synthase-2
*E. granulosus*: *Echinococcus granulosus*
